# Resistance after viral failure on atazanavir-containing therapy: multinational clinical cohort (BMS AI424-128 — 'IMPACT') final analysis

**DOI:** 10.1186/1758-2652-13-S4-P134

**Published:** 2010-11-08

**Authors:** A Zolopa, W Towner, A Lazzarin, G Fätkenheuer, D Butcher, J Uy

**Affiliations:** 1 Stanford University, Palo Alto, CA, USA; 2 Kaiser Permanente, Los Angeles, CA, USA; 3 Ospedale San Raffaele, Milano, Italy; 4 Universität Köln, Cologne, Germany; 5 Bristol-Myers Squibb Research & Development, Rueil-Malmaison, France; 6 Bristol-Myers Squibb Research & Development, Plainsboro, NJ, USA

## Purpose of the study

Real world data on the development of drug resistance after virologic failure (VF) on a protease inhibitor (PI)-based antiretroviral (ARV) regimen are limited. The I50L substitution in protease is the primary mutation associated with atazanavir drug resistance. The primary objective was to compare the prevalence of I50L from VF patients on an unboosted atazanavir (ATV)-based regimen vs. those on a ritonavir-boosted ATV (ATV/r)-based regimen regardless of prior treatment history.

## Methods

IMPACT is a large cross-sectional study of patients with VF while on an atazanavir-containing regimen that was conducted at 220 sites in 8 countries. Demographic/medical information and blood for a genotype resistance test were collected at a single study visit. A substudy evaluated the efficacy of the regimen subsequent to the atazanavir-containing regimen based on when atazanavir was used in therapy.

## Summary of results

IMPACT enrolled 703 patients, and genotype resistance tests were able to be performed for 678. 67 had been on both ATV and ATV/r, and 55 had incomplete ARV histories. Overall, 48/556 evaluable patients had virus with an I50L: 12/96 (12.5%) on ATV and 36/460 (7.83%) on ATV/r (p=0.116). Most patients had been on another PI prior to atazanavir.

88/678 patients were on atazanavir (either ATV or ATV/r) as a first PI, and 69 had a complete ARV history. 3/19 (15.8%) who started ATV and 5/50 (10%) who started ATV/r had an I50L at VF. Phenotype resistance tests were performed for these patients: 55 tests showed full susceptibility while 12 tests showed reduced susceptibility to atazanavir. The viral isolates from these 12 patients remained fully sensitive to both lopinavir and darunavir.

Enrollment for the substudy was below the target enrollment, and sample sizes in all of the comparison groups were too small for meaningful statistical inference.

## Conclusions

In a large clinical cohort of subjects failing atazanavir-based therapy with resistance data, I50L is uncommon in subjects with VF on atazanavir. Most PI-naive subjects failing an atazanavir-based regimen failed with virus susceptible to atazanavir, and all viral isolates were sensitive to the other PIs commonly used to treat virus with PI drug resistance. These real world data provide further support of clinical trial data that have shown preservation of treatment options after VF when atazanavir is used as a first PI.

